# Methylated DNA Recognition during the Reversal of Epigenetic Silencing Is Regulated by Cysteine and Serine Residues in the Epstein-Barr Virus Lytic Switch Protein

**DOI:** 10.1371/journal.ppat.1000005

**Published:** 2008-03-07

**Authors:** Questa H. Karlsson, Celine Schelcher, Elizabeth Verrall, Carlo Petosa, Alison J. Sinclair

**Affiliations:** 1 School of Life Sciences, University of Sussex, Brighton, United Kingdom; 2 EMBL Grenoble, BP 181, Grenoble, France; University of Wisconsin-Madison, United States of America

## Abstract

Epstein-Barr virus (EBV) causes infectious mononucleosis and is associated with various malignancies, including Burkitt's lymphoma and nasopharyngeal carcinoma. Like all herpesviruses, the EBV life cycle alternates between latency and lytic replication. During latency, the viral genome is largely silenced by host-driven methylation of CpG motifs and, in the switch to the lytic cycle, this epigenetic silencing is overturned. A key event is the activation of the viral *BRLF1* gene by the immediate-early protein Zta. Zta is a bZIP transcription factor that preferentially binds to specific response elements (ZREs) in the *BRLF1* promoter (Rp) when these elements are methylated. Zta's ability to trigger lytic cycle activation is severely compromised when a cysteine residue in its bZIP domain is mutated to serine (C189S), but the molecular basis for this effect is unknown. Here we show that the C189S mutant is defective for activating Rp in a Burkitt's lymphoma cell line. The mutant is compromised both *in vitro* and *in vivo* for binding two methylated ZREs in Rp (ZRE2 and ZRE3), although the effect is striking only for ZRE3. Molecular modeling of Zta bound to methylated ZRE3, together with biochemical data, indicate that C189 directly contacts one of the two methyl cytosines within a specific CpG motif. The motif's second methyl cytosine (on the complementary DNA strand) is predicted to contact S186, a residue known to regulate methyl-ZRE recognition. Our results suggest that C189 regulates the enhanced interaction of Zta with methylated DNA in overturning the epigenetic control of viral latency. As C189 is conserved in many bZIP proteins, the selectivity of Zta for methylated DNA may be a paradigm for a more general phenomenon.

## Author Summary

γ herpesviruses are characterized by their life-long persistence in the infected host. This is due in part to their ability to establish latency in infected cells. Epstein-Barr virus (EBV) is almost ubiquitous within the human population. The virus establishes latency in B-lymphocytes and is thought to reactivate and undergo replication following physiological stimuli that lead to B-cell activation. During latency the DNA genome of EBV is methylated, resulting in epigenetic control of viral gene expression. A viral protein that is key to viral reactivation and replication, Zta, is unique amongst transcription factors in displaying enhanced binding to methylated DNA sites. Here, we define the molecular interactions that predispose Zta to interact with methylated binding sites and we identify a mutant of Zta that has lost the preference for methylated sites. This allows us to probe the relevance of the recognition of methylated DNA for the reactivation of EBV from latency and to suggest that EBV has evolved a mechanism to allow it to specifically recognize methylated DNA and overturn epigenetic silencing of its genome imposed by the host.

## Introduction

Methylation of DNA is generally associated with inhibition of gene expression. This is mediated in part by the association of specific methyl-CpG binding proteins with methylated DNA, leading to transcriptional silencing and chromatin remodeling [1], and in part by the inability of some transcription factors to bind to methylated DNA [2]. A notable exception is the bZIP transcription factor Zta (also known as BZLF1, ZEBRA, Z), which displays enhanced recognition for methylated DNA [3],[4].

Zta is encoded by Epstein-Barr virus (EBV), a human herpes virus that infects the majority of the world's population. EBV causes infectious mononucleosis and is linked to malignancies such as endemic Burkitt's lymphoma, nasopharyngeal carcinoma, and Hodgkin's disease [5]. EBV infects then establishes long-term latency in B-lymphocytes [6],[5]. During latency, the EBV genome is heavily methylated and few viral genes are expressed. Disruption of EBV latency is sporadic, characterized by expression of the majority of the EBV gene complement, replication of the genome and release of virus [7],[8].

Zta is the first viral gene expressed during the switch to lytic replication. As well as enhancing its own expression in a positive feedback loop [9], Zta activates a second viral transcription factor, Rta, encoded by *BRLF1* [10]. Both Zta and Rta are essential for viral replication and together promote expression of the remaining viral lytic genes [11]. The *BRLF1* promoter, referred to as Rp, contains three Zta-response elements (ZREs), two of which (ZRE2 and ZRE3) include CpG motifs that are subject to methylation. A pivotal study demonstrated that the interaction of Zta with ZRE2 and ZRE3 is enhanced by methylation [3], a phenomenon believed critical for lytic cycle activation.

The basic region of Zta's bZIP domain contains a cysteine residue, C189, which regulates the redox-sensitivity of DNA-binding activity [12]. Substituting this cysteine with serine (ZtaC189S) is sufficient to prevent reactivation of EBV from latency and EBV genome replication [13],[12]. Here we explore the molecular basis for the dramatic effects of this point mutation. We demonstrate that C189 is critical for the activation of *BRLF1* expression and for recognition of the methylated ZREs in Rp, both *in vivo* and *in vitro*. A previous study had shown that the point mutation S186A compromises Zta's ability to bind both the methylated and non-methylated forms of the ZREs within Rp [4]. We propose a model in which S186 and C189 contact the two cytosine methyl groups of a specific CpG motif, which is conserved between ZRE2 and ZRE3. The relevance of a regulatory role for C189 in methylated DNA recognition by Zta is discussed.

## Results

### C189 in Zta is critical for its ability to activate *BRLF1*

**Figure 1:**
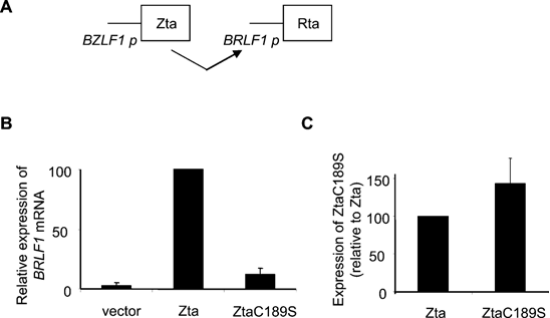
Single amino acid in basic region of Zta blocks the ability to transactivate Rp in BL cells. A. Schematic diagram showing the relationship between Zta expression and activation of the *BRLF1* promoter. B. Expression vectors for Zta, ZtaC189S and the relevant “empty” vector (pBABE) were introduced into Raji cells and their ability to activate the endogenous *BRLF1* gene determined. 24 hours after transfection, RNA was prepared, cDNA was synthesized then amplified using quantitative PCR with specific primers for the *BRLF1* transcript and a housekeeping gene, L32. Expression of *BRLF1* mRNA, following normalization for expression of L32 is shown, relative to that seen following Zta transfection (100%). C. Expression of Zta and ZtaC89S were determined by quantitative PCR and expressed relative to expression of Zta (100%).

We and others have previously shown that altering a single cysteine residue within the DNA contact region of Zta (ZtaC189S) impairs its ability to disrupt EBV latency [13],[12]. Here we asked whether ZtaC189S is competent to initiate one of the earliest events in latency disruption, the transcriptional activation of *BRLF1* (Figure 1A). Expression vectors encoding Zta and ZtaC189S were introduced into a Burkitt's lymphoma derived cell line, Raji, and subsequent transactivation of the endogenous viral Rp was assessed (Figure 1B). Basal expression of *BRLF1* is low in the absence of Zta, but is enhanced 33-fold following Zta expression. In contrast, expression of an equivalent amount of ZtaC189S resulted in only 4-fold enhancement of *BRLF1* expression. Therefore, ZtaC189S is severely compromised for its ability to transactivate the *BRLF1* gene in Raji cells.

### C189 is not essential for the interaction of Zta with non-methylated ZREs

**Figure 2:**
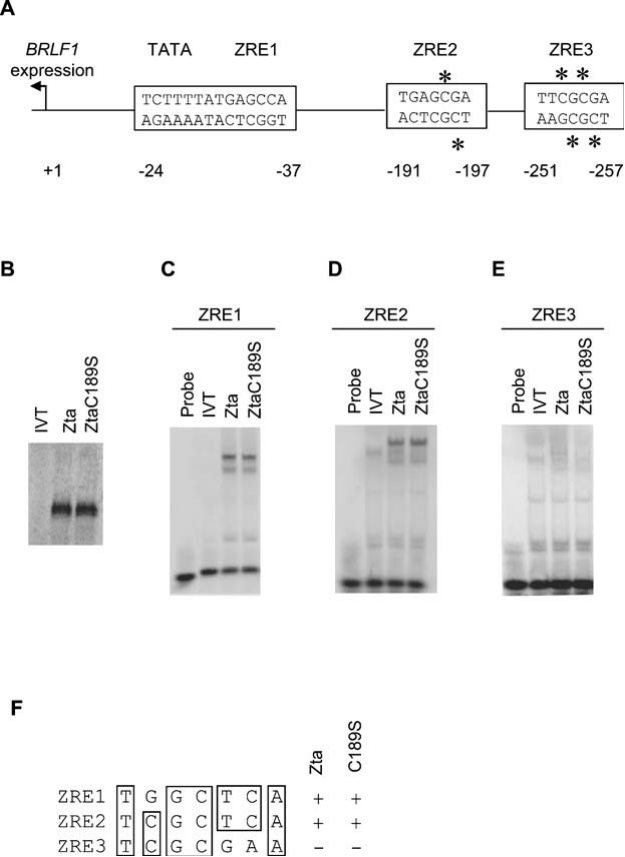
Zta and ZtaC189S interact with non-methylated Rp ZREs equivalently. A. Schematic diagram showing the location of ZREs 1–3 in Rp. Transcription of this gene occurs in a leftwards direction with respect to the viral genome. The numbering relates to the type I EBV genome accession number NC_007605. Asterixes mark the methylated Cytosine residues. B. Zta and Zta C189s were generated in an *in vitro* translation system and fractionated on SDS-PAGE, together with a non-programmed translation reaction (IVT). C.–E. Equivalent amounts of the indicated proteins were subject to EMSA analysis with the probes indicated above. A reaction with no added protein was also included, indicated probe. F. The three ZREs associated with Rp are aligned and their areas of conservation indicated by boxes. The interactions of Zta and ZtaC189S with each site are summarized.

To investigate the molecular basis for this defect, we compared the ability of Zta and ZtaC189S to bind to the three ZREs present in Rp (Figure 2). All three of the ZREs contribute to Rp activity and are important for activation of the endogenous viral gene by chemical stimuli [14], although ZRE1 appears to be dispensable on a fully-methylated template [3]. Zta and ZtaC189S were produced by coupled transcription and translation *in vitro* and their ability to interact with each ZRE was assessed by EMSA (Figure 2). As summarized in Figure 2F, the proteins were equally capable of binding to non-methylated ZRE1 and ZRE2 but, in agreement with previous reports for Zta [3],[12], neither Zta not ZtaC189S showed a detectable interaction with non-methylated ZRE3. Thus, the reduced ability of ZtaC189S to transactivate *BRLF1* is not due to an inherent defect in binding to the ZREs.

### Interaction of Zta with methylated ZREs relies on C189 *in vitro* and *in vivo*

**Figure 3:**
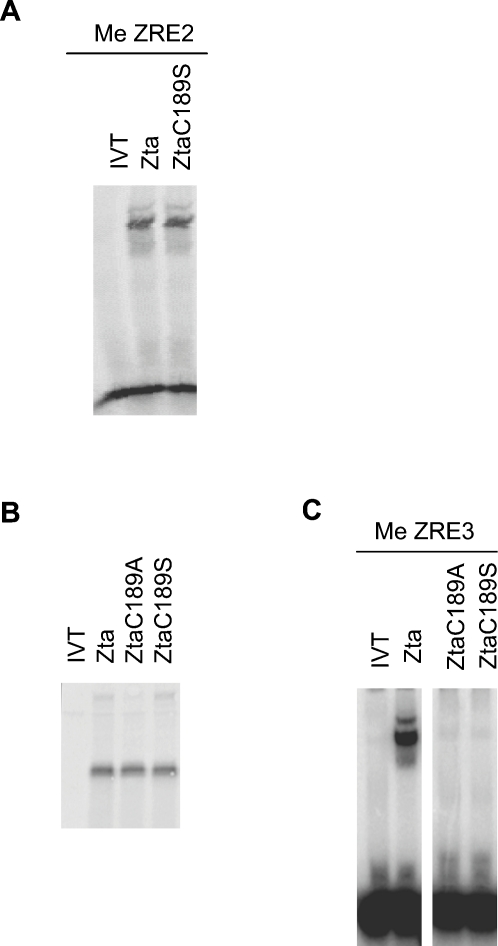
ZtaC189S binding to meZREs in Rp is compromised *in vitro*. A. EMSA analysis was undertaken for meZRE2 with the indicated proteins or an unprogrammed lysate (IVT). B. Zta, ZtaC189A and ZtaC189S were generated *in vitro* and analyzed by SDS-PAGE. C. EMSA analysis of equivalent amounts of the indicated proteins was undertaken with meZRE3 as described above.

We next asked what effect methylation of ZRE2 and ZRE3 had on binding by Zta and ZtaC189S (ZRE1 lacks a CpG motif and hence is not subject to methylation). Note that methylation yields four methyl-cytosines in ZRE3 (two on each strand) but only two in ZRE2 (Figure 2). In line with previous reports, methylation of ZRE3 converted it from a marginal to a strong binding site for Zta and methylation of ZRE2 also enhanced binding (Figure 3 and [3],[12],[4]). Importantly, the ability of ZtaC189S to bind meZRE3 was markedly reduced compared to that of wild-type Zta (Figure 3). Binding of ZtaC189S to meZRE2 was only modestly reduced, to approximately 75% compared to wild type. An independent mutation of C189 to alanine (ZtaC189A) also resulted in decreased binding to meZRE3, suggesting that the decreased binding of the C189S mutant was due to loss of the cysteine and not, for example, merely due to phosphorylation of the newly introduced serine.

**Figure 4:**
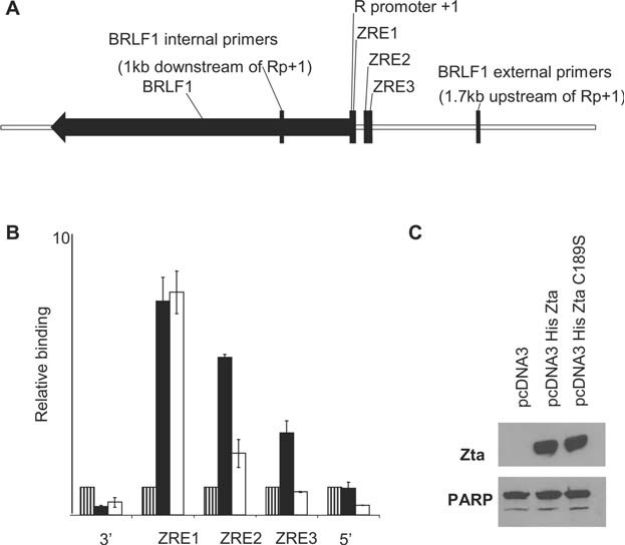
ZtaC189S binding to meZREs in Rp is compromised *in vivo*. A. Schematic representation of the *BRLF1* gene. The black arrow indicates the primary transcript. The location of primer sets used to detect sub-regions of Rp and upstream and downstream regions are indicated relative to the transcription start site. B. HisZta and HisZtaC189S were introduced into 293-BZLF1-KO cells and chromatin prepared. Chromatin affinity purification was undertaken and binding to Rp detected with the indicated primer sets by real-time PCR. The signal was set relative to the “empty vector”, pcDNA3 (striped box), and the signal for Zta (filled box) and ZtaC189S (open box) are shown together with the standard error from duplicate experiments. C. Expression of HisZta, HisZtaC189S and a loading control, PARP, were assessed by western blot analysis.

To substantiate the above findings, we used chromatin precipitation to evaluate the binding of Zta and ZtaC189S to the *BRLF1* promoter *in vivo*. Cells (293-BZLF1-KO) that contain an episomal EBV genome [11] were transfected with vectors encoding either His-tagged Zta or ZtaC189S and equivalence of expression was confirmed by immunoblotting (Figure 4). Zta- and ZtaC189S-associated chromatin complexes were isolated and the co-precipitated DNA was amplified using quantitative PCR with primers spanning the ZREs within Rp (Figure 4). As expected [12], ZRE sequences in the precipitated chromatin were clearly enriched, whereas regions lying 5′ and 3′ of Rp were not. Fine mapping of the chromatin complexes allowed us to differentiate between binding to ZRE1, ZRE2 and ZRE3. Whereas Zta and ZtaC189S bound equally well to ZRE1, the interaction between ZtaC189S and ZRE2 was partly compromised (relative to wild-type Zta) and its interaction with ZRE3 was completely eliminated. As Rp is fully methylated in cells harboring EBV [3], the *in vivo* chromatin association and the *in vitro* DNA-binding analyses correlate well. Taken together, these data demonstrate a critical role for C189 in the interaction of Zta with meZRE3 and, to a lesser extent, meZRE2.

### Methyl-cytosine residues in MeZRE3 required for interaction with Zta

**Figure 5:**
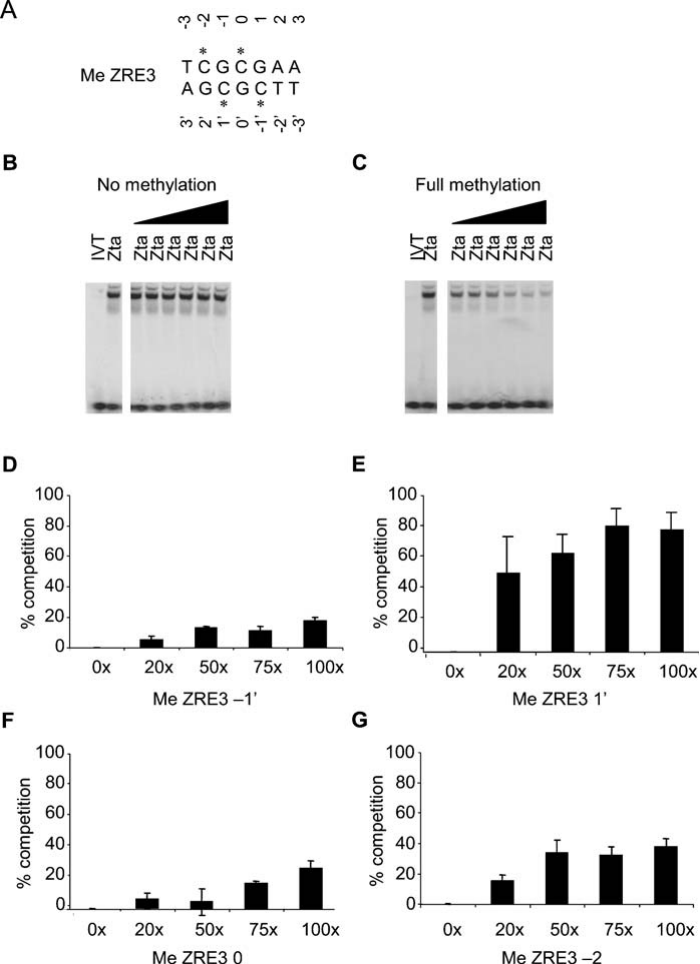
All four methylation sites on ZRE3 contribute to the binding by Zta. A. Schematic representation of the four methyl-cytosine residues in ZRE3. Methylation is indicated by an asterisk and the numbering system is shown. B, C. Competition EMSA reactions were undertaken with a labeled ZRE (ZIIIB) and non-labeled Zta protein. As indicated, increasing amounts (6×, 10×, 20×, 50×, 75× and 100× excess) of unlabelled competitor ZRE3 DNA (methylated or not) was included in the EMSA reaction. D–G. EMSA competition from replicate experiments showing the ability of the indicated excess of each singly methylated ZRE3 site to compete for the binding of Zta. Experiments were undertaken in duplicate and were used to calculate the standard deviation shown in the error bars.

As the interaction of Zta with ZRE3 is methylation-dependent and the site contains four methylated cytosine residues, we explored the relevance of each methylation site (see Figure 5A for numbering convention). A series of double-strand versions of ZRE3 was generated, each containing a single methyl-cytosine, and binding to Zta was assessed in a competition assay. Initial validation of the assay revealed that whereas a 20-fold excess of fully methylated ZRE3 competes efficiently for Zta binding, non-methylated ZRE3 fails to compete, even at a 100-fold excess (Figure 5B,C). Each singly methylated version of ZRE3 competed for binding to some degree (Figure 5D–G) implying that all four methyl groups contribute to the interaction with Zta. The order of competition efficiency was methyl cytosine^1′^>methyl cytosine^−2^>methyl cytosine^0^≥methyl cytosine^−1′^. Thus, methylation within the CpG motif that is common to both ZRE2 and ZRE3 gave stronger competition than within the motif uniquely present in ZRE3.

### Structural modeling rationalizes the methylation dependence of ZRE3 binding

**Figure 6:**
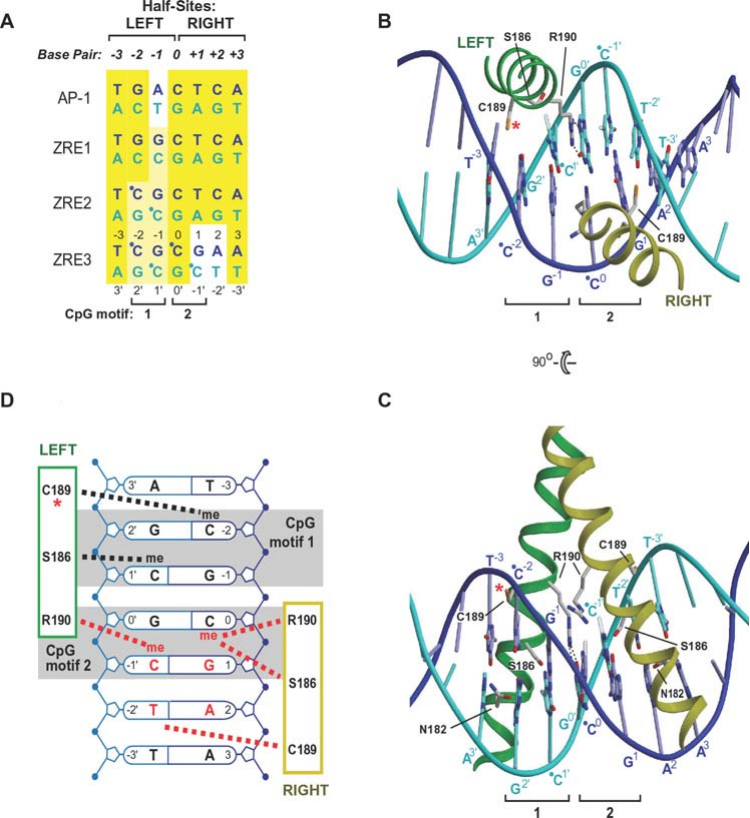
Structural model of Zta bound to meZRE3. A. Alignment of Zta recognition sequences. The numbering convention is shown for base pair positions (bold italics) and individual nucleotides (plain font). Cytosines modified by methylation are indicated by a dot. B. Model of the Zta-meZRE3 complex viewed along the pseudodyad. The methylation sensitive C189 residue (red asterisk) and bidentate hydrogen bond interactions between R190^Left^ and Guanine^0′^ (dotted black lines) are indicated. Cytosine methyl groups are semi-transparent. C. Orthogonal view. The hydrophobic contact between Cytosine^1′^ and S186^Left^ (broken blue line) and hydrogen bond network involving S186^Left^, N182^Left^ and Guanine2′ (dotted black lines) are shown. D. Schematic summary of contacts. van der Waals contacts involving the CpG methyl groups and Zta residues are shown as broken lines.

To better understand the above observations, we modeled the structure of Zta's DNA-binding domain bound to methylated ZRE3 and compared it to the previously reported model of Zta-bound meZRE2 [15]. In the following description, we designate residues within the two Zta monomers and the corresponding DNA half-sites as “Left” or “Right”, and the two CpG motifs as motifs 1 and 2, as summarized in Figures 6A and D. The modeled ZRE2 and ZRE3 structures, both based on the crystal structure of Zta bound to an AP-1 site, differ exclusively in the right half-site, as the two ZREs are identical except at base pairs 1 and 2 (Figure 6A).

In the crystal structure, Zta makes base-specific contacts with the AP-1 site via residues N182 and R190. N182 interacts symmetrically with base pairs ±2 while R190 makes asymmetric contacts: R190^Left^ interacts with the central guanine base while R190^Right^ contacts the DNA phosphate backbone. In the modeled ZRE structures, the contacts mediated by R190 are conserved whereas those by N182 are not – a direct consequence of the DNA sequence difference. More specifically, the N182 contacts are disrupted in the left half-site of ZRE2 (because ZRE2 diverges from AP-1 at base pair –2) and in both half-sites of ZRE3 (divergent at base pairs *±2*). The additional disruption in the right half-site likely explains why Zta binds more weakly to ZRE3 than to ZRE2 ([3]; Figure 2D and E).

**Figure 7:**
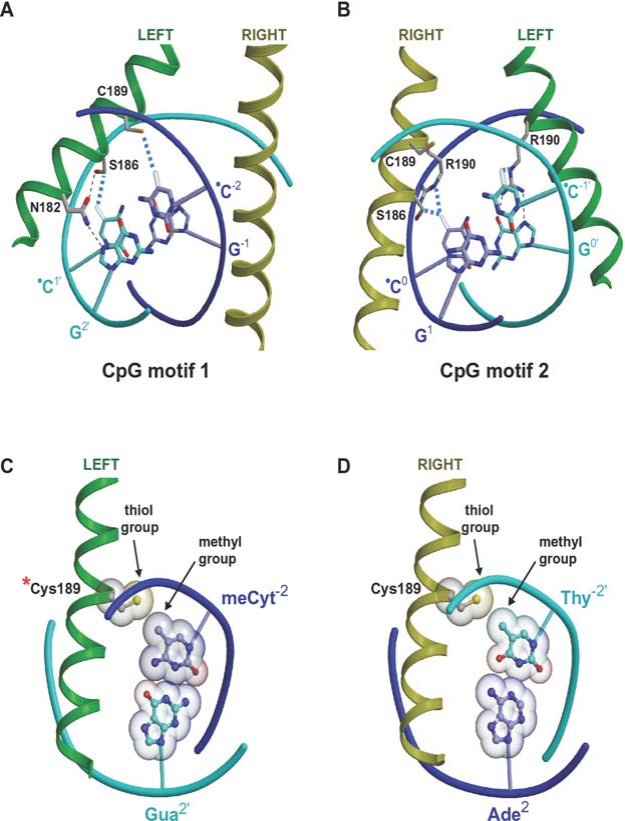
Interactions mediated by the CpG motifs and C189 and S186 residues. A, B. Interactions involving the CpG motifs. Hydrophobic contacts and hydrogen bonds are shown as dashed blue and dotted black lines, respectively. The two motifs are shown in roughly the same orientation. CpG motif 1 interacts exclusively with the Left helix, whereas CpG motif 2 interacts with both bZIP helices. C, D. Thiol-methyl group contacts mediated by C189 residues. Only C189^Left^ (asterisk) can sense CpG methylation status.

How does methylation of ZRE3 enhance Zta binding? The mechanism previously postulated for ZRE2 is that methylation of CpG motif 1 results in a direct contact between the cytosine^1′^ methyl group and S186^Left^, enhancing affinity by stabilizing a hydrogen bond network that involves S186^Left^, N182^Left^ and the CpG motif's Guanosine^2′^ base ([15]; Figures 6C and 7A). The same mechanism can be proposed for ZRE3, as ZRE2 and ZRE3 have identical left half-sites. Also predicted to contribute to enhanced binding is a hydrophobic contact between C189^Left^ and the cytosine^−2^ methyl group (Figure 7A,C). This interaction, not previously described, is common to both the meZRE2- and meZRE3-bound Zta models. Thus, S186^Left^ and C189^Left^ are postulated to interact with CpG motif 1 so as to simultaneously engage the two methyl groups, which are located on complementary DNA strands (Figure 7A). Such interactions are consistent with the ability of ZRE3 singly methylated on cytosine^+1′^ or cytosine^−2^ to compete with fully-methylated ZRE3 for Zta binding (Figure 5).

The ZRE3 model also suggests how methylation of CpG motif 2 influences Zta binding. The cytosine^–1′^ aromatic ring is in a cation-π interaction with the guanidino group of R190^Left^ (Figure 6B and 7B) [16]. Methylation would enhance this interaction by increasing the amount of negative charge in the π-electron system (CH3- is an electron-donating substitutent) and by introducing an additional van der Waals contact with the arginine side chain. On the complementary strand, the Cyt^0^ methyl group contacts both S186^Right^ and R190^Right^. Although R190^Right^ does not interact with a DNA base, S186^Right^ is within hydrogen bond distance of the base-contacting residue N182^Right^; hence, methylation may indirectly influence interactions involving base pair +2 or +3. Although the atomic details cannot reliably be predicted, these probably differ from those previously postulated for S186^Left^ and CpG motif 1, as the stereochemical environments of S186 and N182 differ between the two half-sites. Nevertheless, the prediction that Zta residues contact both methyl groups of CpG motif 2 agrees with the ability of ZRE3 singly-methylated at cytosine^−1′^ or cytosine^0^ to compete with fully methylated ZRE3, albeit more weakly than when methylated within CpG motif 1 (Figure 5). Indeed, the observation that methylation on CpG motifs 1 and 2 has a differential effect on Zta binding is consistent with these motifs mediating non-equivalent interactions with Zta residues, due to their asymmetry relative to the ZRE3 pseudodyad (Figures 6D, 7A and 7B).

### Thiol/methyl-group contacts can explain the decreased ZRE binding affinity of the ZtaC189S mutant

Our modelling predicts that C189^Left^ (marked by an asterisk in Figures 6 and 7) is sensitive to the methylation state of CpG motif 1 via a direct contact involving the residue's thiol group and the cytosine^−2^ methyl group (Figure 7C). This interaction is favored by the hydrophobic nature of the cysteine side chain [17]. Mutation to the more polar serine residue would destabilize this contact by unfavorably juxtaposing the serine hydroxyl and Cyt^−2^ methyl groups. This agrees with the decreased binding affinity observed for ZtaC189S toward both meZRE2 and meZRE3 (Figure 3). Moreover, the absence of this thiol-methyl contact in non-methylated ZRE sites explains why the C189S mutation has little effect on the binding to non-methylated ZRE2 (Figure 2D). A possible alternative explanation, that an altered S186 or R190 conformation causes the decreased affinity, appears unlikely given that C189 contacts neither residue (see, for example, Fig. 6C).

In contrast to C189^Left^, C189^Right^ is too far from any cytosine methyl group to form a direct contact (Figure 6B,C). However, in the ZRE3 model, C189^Right^ is in van der Waals contact with the thymidine^−2′^ methyl group (Figure 7D). This interaction is symmetrically equivalent to that of C189^Left^ with the cytosine^−2^ methyl group and consequently should also be destabilized by the C189S mutation (Figure 6D). The interaction is unique to ZRE3, as the corresponding base in ZRE2 is guanine^−2′^, which lacks a methyl group. Thus, the prediction is that the C189S mutation destabilizes contacts in both half-sites of meZRE3, but in only one of ZRE2. This agrees neatly with the finding that the mutation more severely impairs Zta binding to meZRE3 than to meZRE2 (Figure 3).

### Evidence for a direct contact between C189 and methyl cytosine^−2^

**Figure 8:**
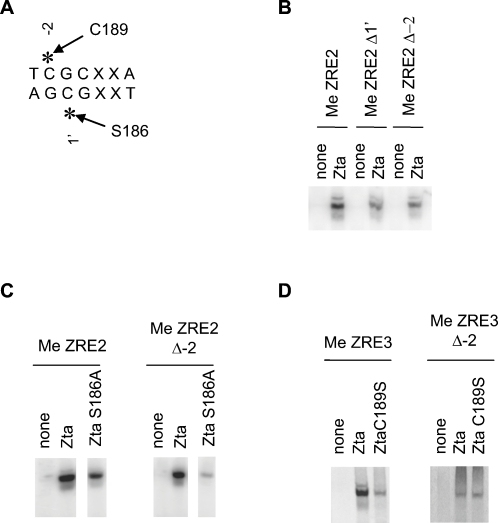
Evidence for C189- and S186- me cytosine^−2^ interactions. A. Contacts between CpG motif 1 and Zta residues. B. EMSA analysis of unprogramed *in vitro* translation reaction (none), or Zta protein with the indicated probes was undertaken. The protein-DNA complex is shown. C. The ability of Zta and ZtaS186A to interact with a probe that omits the methylation of cytosine^−2^ was determined by EMSA. D. The ability of Zta and ZtaC189S to interact with a probe that omits the methylation of cytosine^−2^ was determined by EMSA.

Our modelling predicts that C189 directly contacts the cytosine^−2^ methyl group within CpG motif 1, which is common to ZRE2 and ZRE3. This complements a previous prediction that S186 contacts the cytosine^1′^ methyl group in the same CpG motif [15] (Figure 8A). We tested our hypothesis by assessing the ability of Zta to bind versions of meZRE2 that omit methylation of either the 1′ or −2 cytosine residues. As predicted, omitting either methyl group significantly reduces binding to each (the binding is not entirely abolished, as Zta binds non-methylated ZRE2 with appreciable affinity) (Figure 8B). We next investigated the contribution of the cytosine^−2^ methyl group to the binding affinity of the S186A and C189S point mutants. Our model predicts that omitting this group should more significantly compromise the binding affinity (relative to wild type) of the S186A mutant over that of the C189S mutant, because only the former conserves the cysteine thiol group. As shown in Figure 8C, ZtaS186A binds more weakly to meZRE2 than does wild type Zta, in agreement with previous studies [4]; omitting the cytosine^−2^ methyl group further decreases the relative binding affinity, consistent with the loss of an important contact. This is in stark contrast with the results observed for ZtaC189S: although the relative affinity of this mutant toward meZRE3 is weak, it is not significantly further reduced upon omitting the cytosine^−2^ methyl group (Figure 8D). On the contrary, binding is comparable to that of the wild type, which is dramatically reduced compared to binding to the fully methylated ZRE3; the methyl group of cytosine^−2^ enhances the DNA binding affinity of wild type Zta to a greater extent than that of the C189S mutant. The combined results strongly support the hypothesis that C189 directly contacts the cytosine^−2^ methyl group.

## Discussion

Zta is the only known example of a transcription factor whose binding to specific DNA sequence elements is enhanced by CpG methylation [3]. In other contexts, the distinction between methylated and unmethylated DNA is primarily orchestrated by proteins lacking specific DNA-binding activity, such as the methyl binding proteins MeCP2, MDB1, MDB2, MDB4 and Kaiso [1],[18]. A previous study demonstrated that mutations of S186 affect the ability of Zta to interact with methylated ZRE2 and ZRE3 [4]. However, these mutations also alter interactions with non-methylated ZRE2 and ZRE3 and compromise ZRE1 recognition. In contrast, the C189S mutant is compromised for binding methylated ZRE3, and to a lesser extent ZRE2, but retains wild type affinity towards many unmethylated sites, including ZRE1 (Figures 2 and 3). Thus, the C189S mutant provides a highly selective tool to address the relevance of methylated ZRE recognition for the disruption of latency *in vivo.* Consequently, our results strongly corroborate the hypothesis that Zta binding to methylated ZREs is essential for the reactivation of latent EBV in B-lymphocytes. More specifically, because ZtaC189S is severely compromised for meZRE3 (but not meZRE2) binding, the inability of this mutant to activate *BRLF1* expression in Raji cells suggests that meZRE3 recognition is particularly critical for Rp activation in this cell line (Figure 1).

Our present findings are in apparent disagreement with a previous study showing that the ZtaC189S mutant is only marginally compromised in reactivating *BRLF1* expression [12]. The previous results, which we have verified (data not shown), were obtained using EBV-positive 293 cells (BZLF1-KO-293), an epithelial cell line. In contrast, we observe defective Rp activation in Raji cells, which derive from a Burkitt's lymphoma cell line. EBV infects and replicates in both B-lymphocytes and epithelial cells. However, latency and reactivation from latency *in vivo* is predominantly associated with B-lymphocytes making our findings particularly relevant to understanding how Zta activates Rp in a physiological setting. We speculate that the difference in Zta behavior in B-lymphocytes and epithelial cells may partly reflect differences in the milieu of cellular transcription factors or in chromatin structure. Indeed, a recent study showed that, compared to Raji cells, the level of ZRE2 methylation is slightly lower in BZLF1-ZKO-293 cells, and substantially lower in another epithelial cell line (AGS cells) [4]. Further studies are required to establish whether and how EBV lytic activation is regulated in a cell-specific manner.

Our structural model of Zta bound to meZRE3 reasonably accounts for a number of experimental observations. In particular, the model rationalizes why the binding affinity of Zta for unmethylated ZRE3 is lower than for ZRE2, why methylation increases the binding affinity for both sites, why the C189S mutation compromises methyl-ZRE recognition, and why the latter effect is more pronounced for ZRE3 than for ZRE2. Furthermore, the model suggests that Zta residues contact all four methyl groups in ZRE3's two CpG motifs (Figure 7), consistent with interactions observed for all four singly-methylated ZRE3 variants in a DNA-binding competition experiment (Figure 5). Although unable to explain why methylation within CpG motif 1 yields stronger binding than within motif 2, the model is nevertheless consistent with a differential effect, as it predicts different stereochemical environments for the two motifs.

A key prediction resulting from the model is that a specific C189 residue in the Zta homodimer is sensitive to methylation of the CpG motif common to ZRE2 and ZRE3 by means of a thiol-methyl group contact (Figure 7C). This contact is lost or destabilized by the replacement of C189 by A or S, consistent with the decreased DNA-binding affinity observed for these mutants (Figure 3). In contrast, the homodimer's second C189 residue is predicted to be remote from a CpG motif. In the case of ZRE3, this residue can form a thiol-methyl group contact with a thymine base (Figure 7D). The stability of the latter contact is predicted to be susceptible to the C189S mutation regardless of CpG methylation status. However, the decreased affinity of this mutant can only be observed when the binding to methylated ZRE3 is assessed, as the affinity of wild type Zta for unmethylated ZRE3 is too low to appreciate any further decrease. C189 is implicated in the redox sensitivity of Zta's DNA binding activity, and nitrosylation of this residue has been evoked as one possible mechanism by which nitric oxide down-regulates EBV reactivation [12]. Our results concerning C189 suggest how such regulatory phenomena might potentially be linked to methyl-ZRE recognition and Rp activation.

Our current working model is that S186 and C189 interact with both methyl-cytosines of a specific CpG motif to enhance the binding of Zta to a methylated ZRE, thereby overturning epigenetic silencing of the viral genome. Such methylation-enhanced affinity is conceivably unique to Zta, as no other bZIP proteins are known to posses a serine residue equivalent to S186. On the other hand, C189 is relatively conserved among bZIP proteins: in a sequence alignment of 50 human bZIP proteins, over half conserve this residue, including c-Fos and c-Jun. It is therefore tempting to speculate that CpG methylation may enhance the affinity of certain cellular bZIP proteins for their cognate DNA target sites. This tantalizing potential for Zta's cellular homologs to overturn the epigenetic silencing of genes awaits further investigation.

## Materials and Methods

### Cell culture

293-BZLF1-KO cells [11] and Raji cells were maintained as described previously [13],[12].

### Transfection

Raji cells were transfected as described previously [13],[19]. The sequences of the primers used in determining *BRLF1* gene expression are as follows:

5′-CAGAAAGTCTTCCAAGCCATCC and 5′-CAAACAGACGCAGCCATGA.

Western blot analysis was determined as described previously [19].

### Plasmids

Expression vectors encoding histidine tagged Zta and ZtaC189S were generated by amplifying the coding sequence with the following oligonucleotides, which incorporate a hexa-histidine repeat at the amino terminus and sub-cloned into pcDNA3 (*Invitrogen*) using BamH1 and EcoR1.

5′CTGCACACCGGGGATCCATGCATCATCATCATCATCATATGATGGACCCAAACTCGACTTCT and 5′CTGCACACCGGGGAATTCTTAGAAATTTAAGAGATCCTCGTGTAA

Zta, ZtaC189A, ZtaS186A were generated by site directed mutagenesis.

### Chromatin precipitation

Chromatin was prepared from 293-BZLF1-KO cells 48 hours post transfection [20]. His-tagged protein complexes were isolated on HIS-Select Nickel Affinity Gel slurry *(Sigma-Aldrich)*. Primers adjacent to each ZRE were used to amplify the sites.

ZRE1F (cggctgacatggattactgg);

ZRE1R (tgatgcagagtcgcctaatg);

ZRE2F (cagcagagaggctcggtt);

ZRE2R (tgcaatatttcctccagaaa);

ZRE3f (ggacaagatgtcactcttt);

zre3r (gggaagaaagtatagctac);

Rta3′F (TCCCTGTATTCACTGAGCGTCG);

Rta3′R (GGTCCCTTTGCAGCCAATGC);

Rta 5′F (CTTCGGGATAGTGTTTCAGG);

Rta 5′R (CTCAGCCCGTCTTCTTACC).

### EMSA

Radio labeled probes were generated with [^33^P] or [^32^P] and analyzed as described previously [21],[22],[13],[19]. *In vitro* translated proteins were generated in a rabbit reticulocyte lysate system or a wheat germ translation system, radio labeled with [^35^S] methionine. Competition EMSA was undertaken using unlabeled Zta protein and a radio labeled Zta binding site (ZIIIB).

The 5′ oligonucleotide sequences of the ZRE1, 2 and 3 probes (*Invitrogen*) and their methylated versions, with methyl-cytosine marked as O, (*Sigma*) are as follows:

ZRE1:5′-GATCTCTTTTATGAGCCATTGGCA-3′

ZRE2:5′-GATCATAAAATCGCTCATAAGCTT-3′

ZRE3:5′-GATCTATAGCATCGCGAATTTTGA-3′

ZRE2-meth:5′-GATCATAAAATOGCTCATAAGCTT-3′

ZRE3-meth:5′-GATCTATAGCATOGOGAATTTTGA-3′

Δ1′ or Δ-2 versions of the methylated primers were also synthesized (*Sigma*).

### Molecular modeling

The crystal structure of Zta bound to a 19-mer DNA duplex containing an AP-1 site (PDB accession id 2C9L) was used to model Zta bound to a methylated ZRE3 site. Base-pair replacements converting an AP-1 sequence to that of ZRE3 (see Figure 4A) were made in program O [23], with bases set to adopt ideal Watson-Crick base-pairing and the template DNA backbone kept fixed. Methylation at a given C:G base pair position was modeled by least-squares superposition of a m^5^C:G base pair taken from the crystal structure of the self-complementary DNA duplex CCAGGC(m^5^C)TGG [24]; PDB accession id 2D25. Figures 6 and 7 were prepared using Bobscript [25] and Raster3d [26].

### Accession numbers

EBV genome used type 1 NC_007605; Zta (BZLF1) swiss prot P03206; Burkitt's lymphoma OMIM # 113970.
